# Supplementary material for: Characterization of Inducible HSP70 Genes in an Antarctic Yeast, Glaciozyma antarctica PI12, in Response to Thermal Stress
Source: Microorganisms. 2021 Sep 30;9(10):2069. doi: 10.3390/microorganisms9102069 (PMC8540855; doi:10.3390/microorganisms9102069)
Supplement: Supplementary file 1 [file microorganisms-09-02069-s001.zip › microorganisms-1328239-supplementary.pdf]

microorganisms

Supplementary Materials

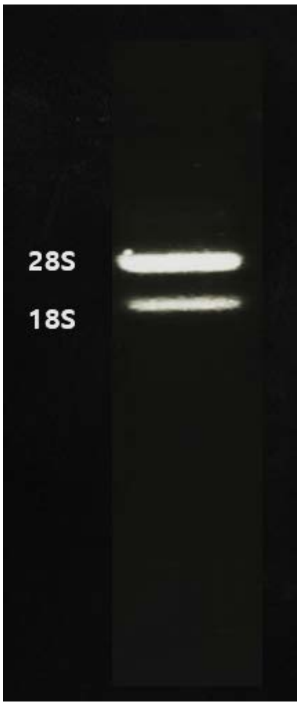

Figure S1. RNA Extraction Result. 1% agarose gel electrophoresis of total RNA extracted from *G. antarctica*.

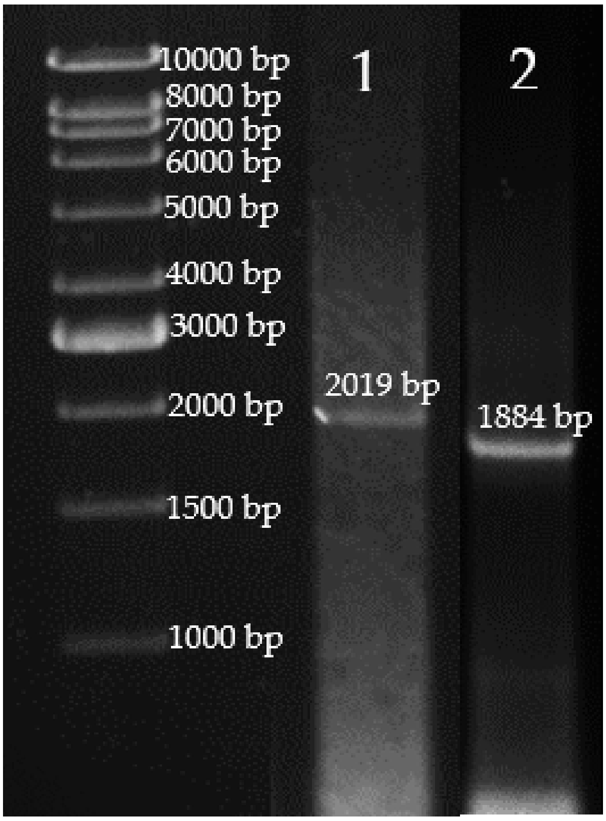

Figure S2. DNA Amplification of Hsp70 genes from G. antarctica. 1% agarose gel of amplified *Hsp70* genes from *G. antarctica* total RNA. 1) *GaHsp70-2*; 2) *GaHsp70-1*.

Table S1. Identity between amino acid sequences of heat shock proteins obtained from *G. antarctica* PI12.

Gene target	Closest database match	Query cover (%)	Identity (%)	E-value	Genbank accession number
GaHsp70-1	Heat shock cognate 70 [*Rhodotorula graminis* WP1]	93	91.75	0	XP_018267745.1
	Hsp71-like protein [*Rhodotorula diabovata*]	93	91.58	0	TNY21258.1
	heat shock cognate 70 [*Rhodotorula* sp. JG-1b]	94	89.2	0	KWU41802.1
	70kDa heat shock protein [*Rhodotorula mucilaginosa*]	94	89.03	0	KAG0657616.1
GaHsp70-2	Heat shock protein 70 family [*Leucosporidium creatinivorum*]	99	89.73	0	ORY92759.1
	Hsp70-like protein [*Microbotryum lychnidis-dioicae* p1A1 Lamole]	99	87.2	0	KDE08371.1
	Molecular chaperone DnaK [*Rhodotorula toruloides* NP11]	99	83.06	0	XP_016269663.1
	Chaperone protein DnaK [*Puccinia sorghi*]	99	73.81	0	KNZ63140.1

```
GaHsp70-1        1 -----------------------------------MTTPGKAIGIDLGTTYSCVGVWQND
GaHsp70-2        1 MLSAARISARSVRPSTPFATGRSLQSLQAHVRHNSSKVSGPVIGIDLGTTNSCVAIMEGK
Saccharomyces    1 --------------------------------------MSKAVGIDLGTTYSCVAHFAND
Arabidopsis      1 ---------------------------------MAGKGEGPAIGIDLGTTYSCVGVWQHD
Drosophila       1 ---------------------------------------MPAIGIDLGTTYSCVGVYQHG
Homo             1 ---------------------------------------MSVVGIDLGFQSCYVAVARAG

GaHsp70-1       26 RVEIIANDQGNRTTPSYVGFT-DNERLIGDAAKNQTAMNPHNTVFDAKRLIGRKFADAEV
GaHsp70-2       61 IPRVIENAEGGRTTPSVVAFTKDGERLVGLPAKRQGVVNHENTFFATKRLIGRKYTDAEV
Saccharomyces   23 RVDIIANDQGNRTTPSFVAFT-DTERLIGDAAKNQAAMNPSNTVFDAKRLIGRNFNDPEV
Arabidopsis     28 RVEIIANDQGNRTTPSYVAFT-DSERLIGDAAKNQVAMNPTNTVFDAKRLIGRRYSDPSV
Drosophila      22 KVEIIANDQGNRTTPSYVAFT-DSERLIGDPAKNQVAMNPRNTVFDAKRLIGRKYDDPKI
Homo            22 GIETIANEYSDRCTPACISFG-PKNRSIGAAAKSQVISNAKNTVQGFKRFHGRAFSDPFV

GaHsp70-1       85 QSDMKHWPFKIIDKAT-KPIIQVEYRGETKEFTPEEISSMILLKMKETAEAYLGTVVTNA
GaHsp70-2      121 QKDINNVPFKIVKHTN----GDAWLEARGQKYSPSQIGAFVVGKMKETAEGYLGRTVKHA
Saccharomyces   82 QADMKHFPFKLIDVDG-KPQIQVEFKGETKNFTPEQISSMVLGKMKETAESYLGAKVNDA
Arabidopsis     87 QADKSHWPFKVVSGPGEKPMIVVNHKGEEKQFSAEEISSMVLIKMREIAEAFLGSPVKNA
Drosophila      81 AEDMKHWPFKVVSDGG-KPKIGVEYKGESKRFAPEEISSMVLTKMKETAEAYLGESITDA
Homo            81 EAEKSNLAYDIVQWPTGLTGIKVTYMEEERNFTTEQVTAMLLSKLKETAESVLKKPVVDC

GaHsp70-1      144 VVTVPAYFNDSQRQATKDAGVISGMNVLRIINEPTAAAIAYGLDK--K---TVGEKNVLI
GaHsp70-2      177 VVTVPAYFNDSQRQATKDAGTIAGLDVLRVINEPTAAALAYGLDR-------TDTRLIAV
Saccharomyces  141 VVTVPAYFNDSQRQATKDAGTIAGLNVLRIINEPTAAAIAYGLDK--K---GK-EEHVLI
Arabidopsis    147 VVTVPAYFNDSQRQATKDAGVISGLNVMRIINEPTAAAIAYGLDKKAS---SVGEKNVLI
Drosophila     140 VITVPAYFNDSQRQATKDAGHIAGLNVLRIINEPTAAALAYGLDKN-----LKGERNVLI
Homo           141 VVSVPCFYTDAERRSVMDATQIAGLNCLRLMNETTAVALAYGIYKQDLPRLEEKPRNVVF

GaHsp70-1      199 FDLGGGTFDVSLLTIEEG-IFEVKATAGDTHLGGEDFDNRLVTHFVGEFKRKNKKDLSTN
GaHsp70-2      230 YDLGGGTFDVSILEMQNG-VFEVKSTNGNTHLGGEDFDIELVNYIVAEFKKESGLDLSKD
Saccharomyces  195 FDLGGGTFDVSLLSIEDG-IFEVKATAGDTHLGGEDFDNRLVNHFIQEFKRKNKKDLSTN
Arabidopsis    204 FDLGGGTFDVSLLTIEEG-IFEVKAAG-DTHLGGEDFDNRMVNHFVQEFKRKNKKDITGN
Drosophila     195 FDLGGGTFDVSILTIDEGSLFEVRSTAGDTHLGGEDFDNRLVTHLADEFKRKYKKDLRSN
Homo           201 VDMGHSAYQVSVCAFNRG-KLKVLATAFDTTLGGRKFDEVLVNHFCEEFGKKYKLDIKSK

GaHsp70-1      258 ARALRRLRTACERAKRTLSS-AAQTTIEIDSLFEG---------------------DLFR
GaHsp70-2      289 RMAIQRIREAAEKAKIELSS-TTQTDINLPYITADASGPKHVNMKMSRSQLEQLVSKLIS
Saccharomyces  254 QRALRRLRTACERAKRTLSS-SAQTSVEIDSLFEGID----FYTSITRARFEELCADLFR
Arabidopsis    262 PRALRRLRTACERAKRTLSS-TAQTTIEIDSLFEGID----FYTTITRARFEELNMDLFR
Drosophila     255 PRALRRLRTAAERAKRTLSS-STEATIEIDALFEGQD----FYTKVSRARFEELCADLFR
Homo           260 IRALLRLSQECEKLKKLMSANASDLPLSIECFMNDVD----VSGTMNRGKFLEMCNDLLA

GaHsp70-1      296 STMEPVEKVLKDSKIDKGNVHEIVLVGGSTRIPRVQKLVSDFFNGKEPNKSINPDEAVAY
GaHsp70-2      348 ETVDPCKKAISDAGVKASEINEVILVGGMSRMPRVIDTVKAVFG-RDPSKGVNPDEAVAV
Saccharomyces  309 STLDPVEKVLRDAKLDKSQVDEIVLVGGSTRIPKVQKLVTDYFNGKEPNRSINPDEAVAY
Arabidopsis    317 KCMEPVEKCLRDAKMDKSSVHDVVLVGGSTRIPKVQQLLQDFFNGKELCKSINPDEAVAY
Drosophila     310 NTLQPVEKALNDAKMDKGQIHDIVLVGGSTRIPKVQSLLQDFFHGKNLNLSINPDEAVAY
Homo           316 RVEPPLRSVLEQTKLKKEDIYAVEIVGGATRIPAVKEKISKFFG-KELSTTLNADEAVTR

GaHsp70-1      356 GAAVQAAILTGDTSEKTQDLLLLDVAPLSTGIETAGGVMTKLIPRNTTVPTKKSETFSTY
GaHsp70-2      407 GASIQGGVLAG----SVTDVLLLDVTPLSLGIETLGGVFTRLINRNTTIPTKKSQTFSTA
Saccharomyces  369 GAAVQAAILTGDESSKTQDLLLLDVAPLSLGIETAGGVMTKLIPRNSTIPTKKSEIFSTY
Arabidopsis    377 GAAVQAAILSGEGNEKVQDLLLLDVTPLSLGLETAGGVMTVLIPRNTTIPTKKEQIFSTY
Drosophila     370 GAAVQAAILSGDQSGKIQDVLLVDVAPLSLGIETAGGVMTKLIERNCRIPCKQTKTFSTY
Homo           375 GCALQCAILSPAFKVREFSITDVVPYPISLRWNSPAEEGSSDCEVFSKNHAAPFSKVLTF
```

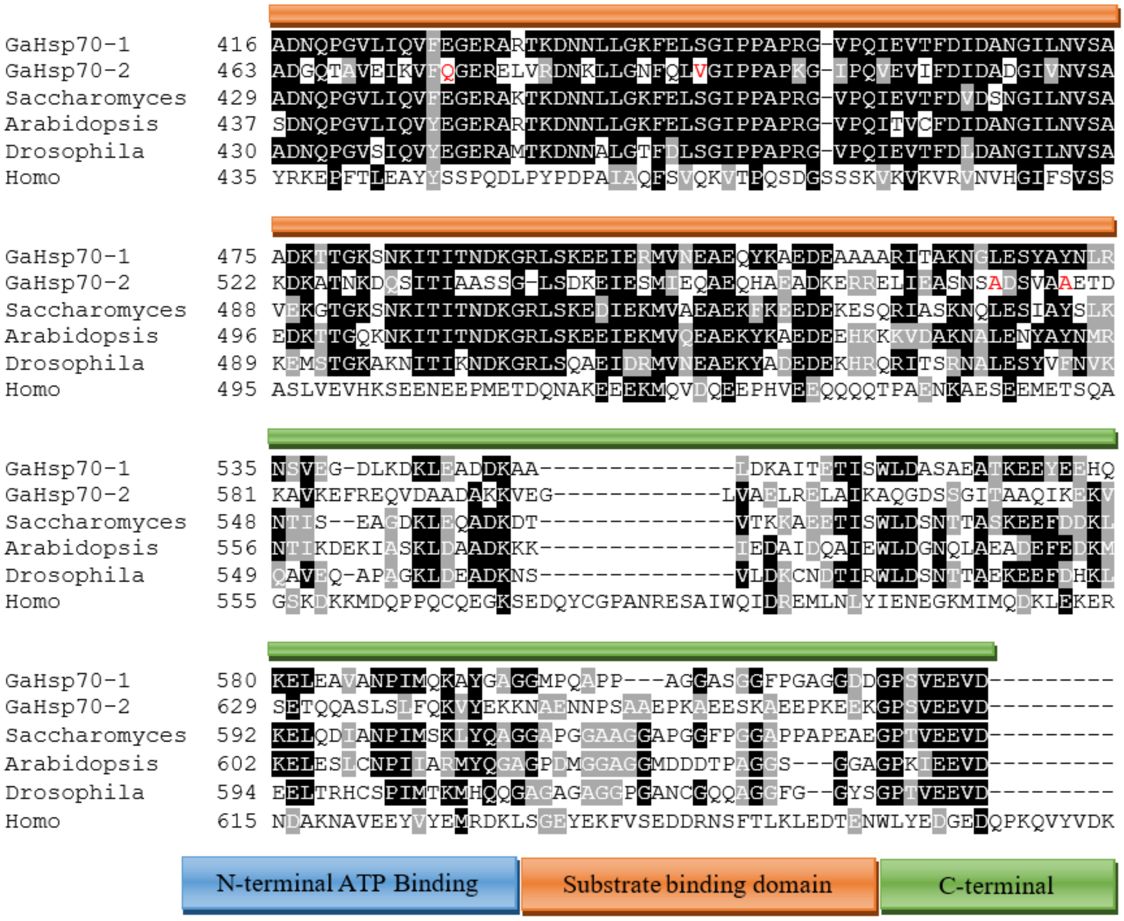

Figure S3. Multiple sequence alignment and domain analysis of *G. antarctica* HSP70s aligned with HSP70 proteins from *S. cerevisiae Ssa1* (GenBank Accession: GHM92458.1), *Arabidopsis thaliana* (GenBank Accession: NP_187864.1), *Drosophila melanogaster* (GenBank Accession: AAK30248.1) and *Homo sapiens* (GenBank Accession: AAC50076.1). The red letter in the alignment showed the residue substitutions to Alanine, less polar residues and charged residues mostly at the N-terminal ATP binding site.

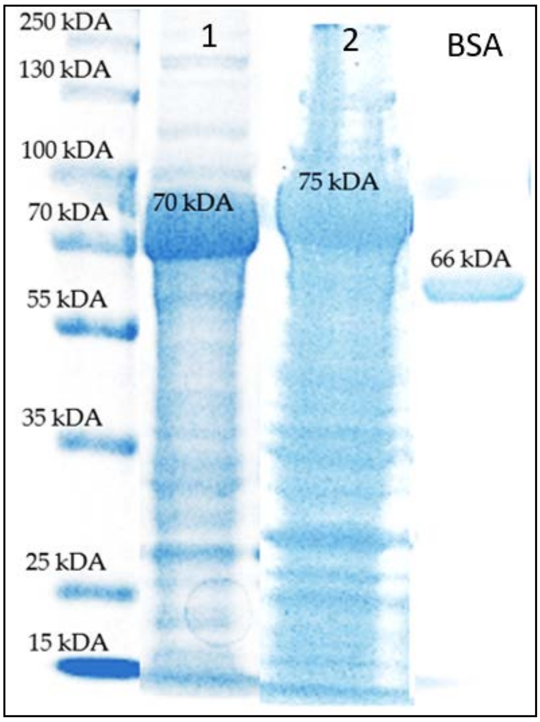

Figure S4. Protein expression of HSP70 proteins from G. antarctica. SDS-PAGE of *G. antarctica* HSP70 protein expression at 20 °C. Lane 1: GaHSP70-1, Lane 2: GaHSP70-2. BSA with the size of 66 kDa was ran as a control.

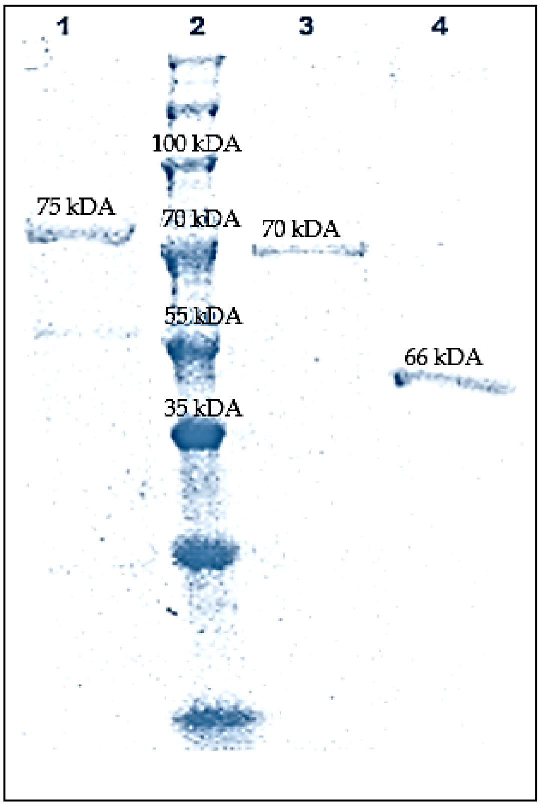

Figure S5. Purified protein of HSP70 proteins from *G. antarctica*. SDS-PAGE of *G. antarctica* HSP70 purified protein ran at 1 mg/mL. Lane 1: GaHSP70-2; Lane 2: Protein marker; Lane 3: GaHSP70-1 and Lane 4: BSA.
